# The potential of general practice to support young people who self-harm: a narrative review

**DOI:** 10.3399/BJGPO.2021.0159

**Published:** 2022-02-09

**Authors:** Faraz Mughal, Lisa Dikomitis, Opeyemi O Babatunde, Carolyn A Chew-Graham

**Affiliations:** 1 School of Medicine, Keele University, Keele, UK; 2 Unit of Academic Primary Care, Warwick Medical School, University of Warwick, Coventry, UK; 3 Midlands Partnership NHS Foundation Trust, Stafford, UK; 4 National Institute for Health Research Applied Research Collaboration West Midlands, Coventry, UK

**Keywords:** adolescent, research methods (other), mental health, general practice

## Abstract

**Background:**

Self-harm in young people is a growing public health concern. Young people commonly present to their GP for help with self-harm, and thus general practice may be a key setting to support young people who have self-harmed.

**Aim:**

To examine the potential of general practice to support young people aged 10–25 years who have harmed themselves.

**Design & setting:**

A narrative review of published and grey literature.

**Method:**

The Scale for the Assessment of Narrative Review Articles (SANRA) was used to guide a narrative review to examine the potential of general practice to support young people who have self-harmed. The evidence is presented textually.

**Results:**

The included evidence showed that GPs have a key role in supporting young people, and they sometimes relied on gut feeling when handling uncertainty on how to help young people who had self-harmed. Young people described the importance of initial clinician responses after disclosing self-harm, and if they were perceived to be negative, the self-harm could become worse.

**Conclusion:**

In context of the evidence included, this review found that general practice is a key setting for the identification and management of self-harm in young people; but improvements are needed to enhance general practice care for young people to fulfil its potential.

## How this fits in

The potential of general practice to support young people who have harmed themselves has, to the authors’ knowledge, not been examined. This comprehensive narrative review found that general practice is a key setting in the identification and management of self-harm in young people. The improvements needed in clinical practice and priorities for future research are outlined for general practice to fulfil its potential.

## Introduction

Self-harm is a public health priority and is the strongest risk factor for suicide.^1^ Self-harm, defined as self-injury or self-poisoning irrespective of suicidal intent, is common in young people; for example, 26% of females aged 16–24 years have reported previously harming themselves, and this is likely to be an underestimation.^2,3^ Self-harm in young people is a result of a complex interplay between genetic, biological, cultural, psychiatric, psychological, and social factors.^4^ Feelings of hopelessness, distress, and impulsivity are closely associated with self-harm in young people, and self-harm can lead to future repeat self-harm, depression, anxiety, and poor education and employment outcomes.^5,6^

In the UK, young people who self-harm seek help most often (25%) through general practice.^7^ In adolescents aged 12–17 years in England, self-poisoning is the most common form of self-harm (71%) presenting to hospital, and in the community self-cutting is predominant (89%).^8^ Rates of self-harm observed in young people’s general practice records have increased over recent years, with the peak occurrence of overdose on anti-inflammatory, antidepressant, and opioid medications in young people to be 16–18 years in females, and 19–24 years in males.^9^

The UK’s National Institute for Health and Care Excellence (NICE) clinical guidelines for self-harm identifies primary care as an important setting for the management of self-harm, but only a few guideline recommendations are specific to general practice.^2^ A key setting of support for young people who self-harm is general practice; however, its potential to support young people remains unexplored. The aim of this narrative review was to examine the potential of general practice to support young people (aged 10–25 years) who had self-harmed.

## Method

A search was conducted of literature published from 2011–2021 in Google Scholar and PubMed on 10 August 2021. Search terms included ‘self-harm’, ‘young people’, ‘general practice’, and ‘primary care’ combined with ‘AND’ Boolean operator. Inclusion criteria were published studies of any design and grey literature reporting primary data addressing the aim of this review over the past 10 years. The exclusion criteria were: non-research manuscript publications, data on young people (10–25 years) not explicitly reported, and literature not in the English language. Titles and abstracts of search findings were screened for eligibility by one author, and full text of studies or reports were included if eligible. References of relevant literature were hand-searched.

One author (FM) read and reread included studies and summarised the findings from studies. The three remaining authors commented extensively on findings and supported the refining and critical interpretation of key study results. The authors have professional backgrounds in social science, medical anthropology, evidence synthesis, applied health research, and general practice, which increases the trustworthiness and credibility of review findings.^10^

The conduct of this review was guided by the SANRA because narrative reviews are better suited to addressing broad topics.^11^ All authors were involved in review conception and design, and interpretation of review findings.

## Results

The literature search generated 328 unique citations, of which eight studies were included in this review. The findings from these studies are textually presented below.

In a qualitative study of GPs in England, Michail and Tait described how GPs exhibited uncertainty when managing young people who had self-harmed and relied on *‘* *gut feeling’*: The authors interviewed 28 GPs from the East Midlands, UK, and included a description of researcher reflexivity.^12^

A systematic review, including 789 GPs across Europe, Australia, and the US, on the role of the GP in the management of self-harm, found that some GPs *‘* *see themselves as a frontline service for young people who self-harm* *’*, but as identified by Michail and Tait, some GPs lacked confidence and wanted training on assessing young people in the consultation.^13^ This review had good overall methodological quality, but only included studies from high-income countries. The authors identified that the involvement of parents and carers of young people was a facilitator for GP management, but such parental involvement was also a barrier, owing to its potential to hinder an open discussion of self-harm in the GP consultation.^13^

In a mixed-methods study by Fox *et al* on GPs’ role in identifying young people who self-harm, the authors found GPs wanted practical information on self-harm.^14^ However, a small number of GPs (*n* = 28) completed the survey in this study, and therefore this may not be the view of other English GPs. A recent survey of 178 GPs in Ireland found that GP training for youth mental health and self-harm resulted in greater GP empathy and confidence on knowledge of self-harm in young people.^15^

Bailey *et al* undertook participatory action research with young people, GPs, and practice nurses in a mixed-methods study, and described young people’s concerns around disclosing their self-harm to a GP: *‘I was scared to talk to the doctor … I just didn’t feel confident enough’*; and their suggestions at helpful ways for clinicians to respond to self-harm: *‘say to you no matter what you’re going through there is people there that can help’.* ^16^ Practice nurses said they relied on GPs if a young person disclosed self-harm to them.^16^

In an interview study of young people (aged 19–25 years) in England about their experiences of self-harm care in general practice, the authors identified three main themes from reflexive thematic analysis: help-seeking avenues, barriers to seeking help from general practice, and facilitators to accessing care.^17^ Young people stated the importance of GP responses to self-harm disclosure and described how these influenced future help-seeking: *‘*… *I felt I needed to tell him* […] *that I’m actually overdosing on them* [antidepressants] … *I did tell him, and once again I didn’t get any reaction* […] *so I decided to stop my medication without telling him* […] *I’ve never been to see the GP since and it’s been six months (Lucy, 24 years).’* Young people highlighted that relationship-based care with GPs who listen and offer follow-up was a key facilitator in accessing general practice care.^17^ This study was informed by patient and public involvement at each stage; however, it was limited by its convenience sample, and therefore the findings may not be transferable to all young people who have self-harmed.These findings are similar to what young people described in an Australian context, albeit from two focus groups of only 10 young people in total; for example, young people stated they wanted collaborative dialogue with their GP around self-harm disclosure and mentioned the importance of GP attitudes to self-harm, and how negative responses could *‘* *possibly even exacerbate self-harming behaviours* *’*.^18^

Morgan *et al* found in an observational general practice electronic health record study using the Clinical Practice Research Datalink (CPRD) that young people (aged 10–19 years) from more deprived areas were 23% less likely to be referred to mental health services in the first year after recorded self-harm compared with general practices in less deprived areas.^19^ This is concerning because self-harm incidence in young people was highest in the most deprived areas. The use of the CPRD for this study enabled a broad representation of self-harm episodes presenting to UK general practice; however, as this database depends solely on the accuracy of self-harm clinical coding, episodes of self-harm may therefore be underestimated.

## Discussion

This review highlights important implications for international primary care practice and policy, and considerations for future research. GPs and primary care physicians require ongoing professional training on self-harm knowledge and management in young people, including navigating the involvement of parents and carers, and use of remote consultations for self-harm within a general practice context. In established primary care systems, there may be the implementation of remote consultations within routine GP care moving forward, and thus there is a need for evidence-informed guidelines for GPs and family medicine doctors when managing young people who self-harm remotely.

There is also the need for GPs to tailor communication with young people who have self-harmed, which can support future help-seeking and prevent negative consequences of a poorly perceived consultation by young people, which is key in preventing repeat self-harm and lowering suicide risk.

GPs must be supported to establish continuity of care with an individual young person who has self-harmed. General practices can become young-people friendly (for example, through improving digitalisation of access), and appointments can be flexibly offered to help young people access care. Morgan *et al* highlighted that the inverse care law is present in the management of self-harm in young people in general practice in more deprived areas, and the inverse care law has been noted to be *‘* *a continuing blot on the record of the National Health Service* *’*.^19–21^ Furthermore, it is documented that the inverse care law persists in low-income and middle-income countries:in this review, studies were included that contained evidence from high-income countries, and therefore self-harm in young people identified in primary care and community settings outside these countries requires the availability of specific specialist support.^22^

Timely access to psychological services for young people who self-harm is key in access to early therapies for self-harm, and third-sector organisations can have a supporting role in helping GPs manage young people in the community, closer to home.^23^ In addition to GPs and family medicine doctors undertaking early identification and intervention of self-harm in young people in general practice, GPs can facilitate the holistic management of young people receiving specialist mental health care through shared communication. They can also support the follow-up care of young people who are discharged from mental health services.^13^

There have been clinically significant levels of mental distress detected early in the COVID-19 pandemic of around 10%, particularly in young people aged 18–24 years.^24^ Episodes of self-harm recorded in general practice, including young adults, have been reported to have not risen during the COVID-19 pandemic. However, the identified increase in distress is important, namely because it is a known risk factor for self-harm, and in turn emphasises the role general practice has in supporting young people who are distressed, and in preventing self-harm behaviour.^25^

In the context of the limited evidence base, future research in this area with patient and public involvement is crucial to inform clinical practice and policy. It is important to better understand how GPs manage young people who self-harm to establish current practice, including through remote consultations, and gain perspectives on acceptable treatments from young people, their families, and GPs. Further qualitative research using a longitudinal design with young people who have self-harmed would allow understanding of critical moments and changes in self-harm to be captured over time.

At the time of writing, the COVID-19 pandemic continues and while vaccination programmes have made progress, new variants are emerging, and young people remain susceptible to COVID-19. Therefore, it is important that general-practice recorded episodes of self-harm in young people are monitored to help shape the primary care response.

Finally, research is needed to develop and evaluate general practice-based interventions for self-harm in young people to address the intervention evidence gap. This may facilitate early intervention to reduce repeat self-harm and distress in young people and families, and potentially reduce healthcare costs.

In conclusion, at present, there is great potential for general practice to support and manage young people who have harmed themselves to help reduce repeat self-harm and provide ongoing self-harm care. However, this review identified that improvements are needed to enhance general practice care for young people with self-harm behaviour, particularly in a COVID-19 context, to fulfil the potential of general practice.
